# Supplementary material for: Comparative Transcriptome Analysis of the Expression of Antioxidant and Immunity Genes in the Spleen of a Cyanidin 3-O-Glucoside-Treated Alzheimer’s Mouse Model
Source: Antioxidants (Basel). 2021 Sep 9;10(9):1435. doi: 10.3390/antiox10091435 (PMC8472539; doi:10.3390/antiox10091435)
Supplement: Supplementary file 1 [file antioxidants-10-01435-s001.zip › supplimentary_Figures.pdf]

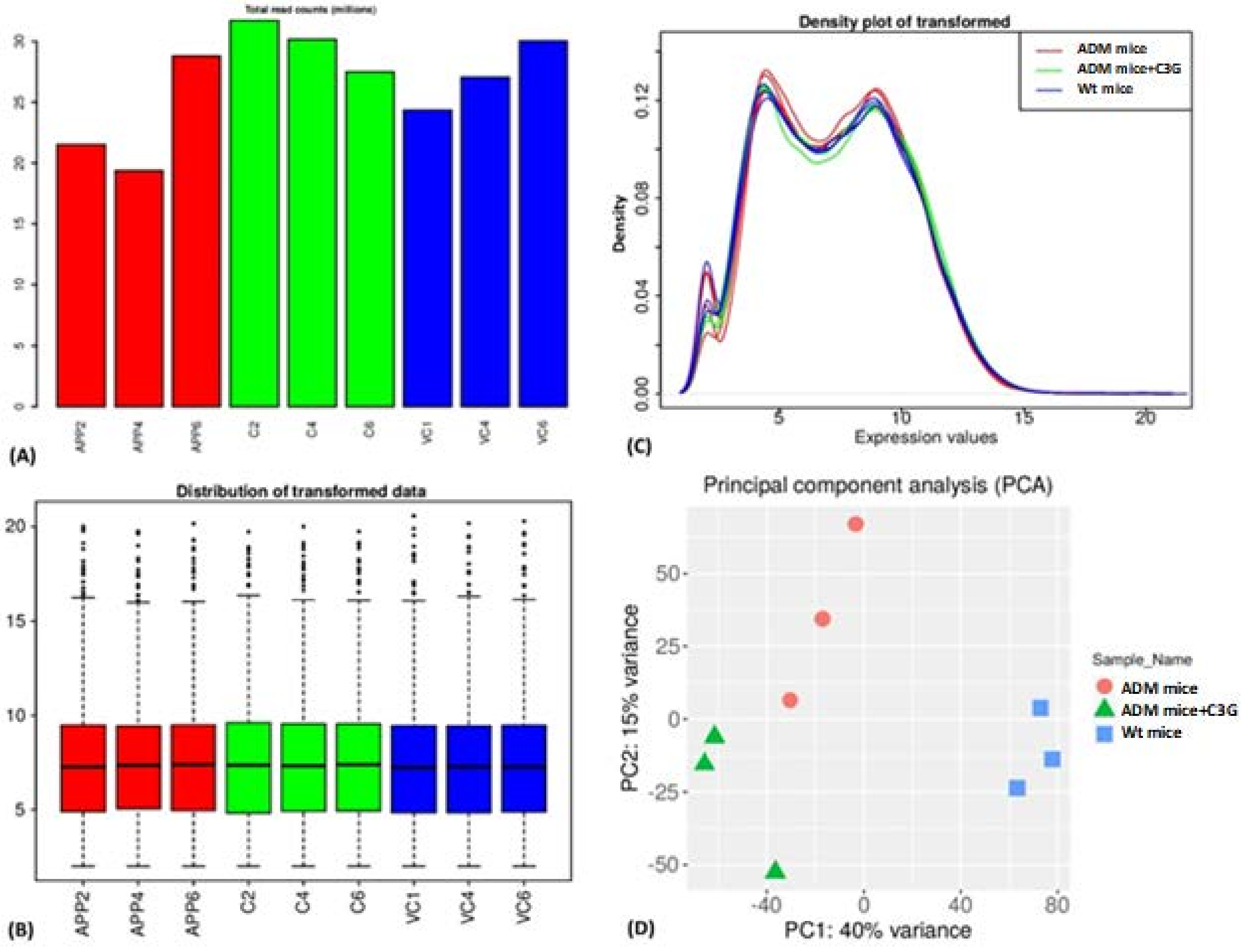

Figure S1a**.** Graphical representation of preprocessing results. (A) Bar plot of total read count in all nine samples. (B) Density plot of transformed data. (C) distribution of transformed data. (D) PCA plots of differential expression against the first and the second principal components were used to determine the difference between the groups.

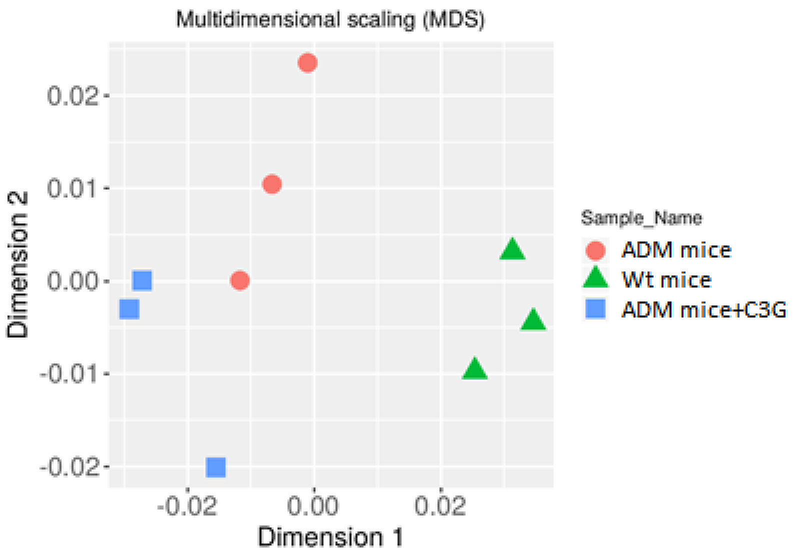

A

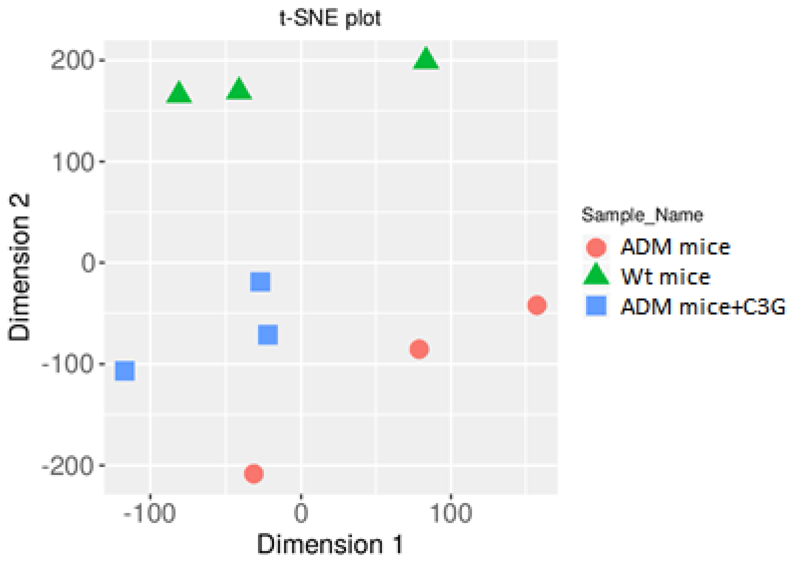

B

Figure S1b. Multidimensional scaling and t-SNE plot for all sample used in the study

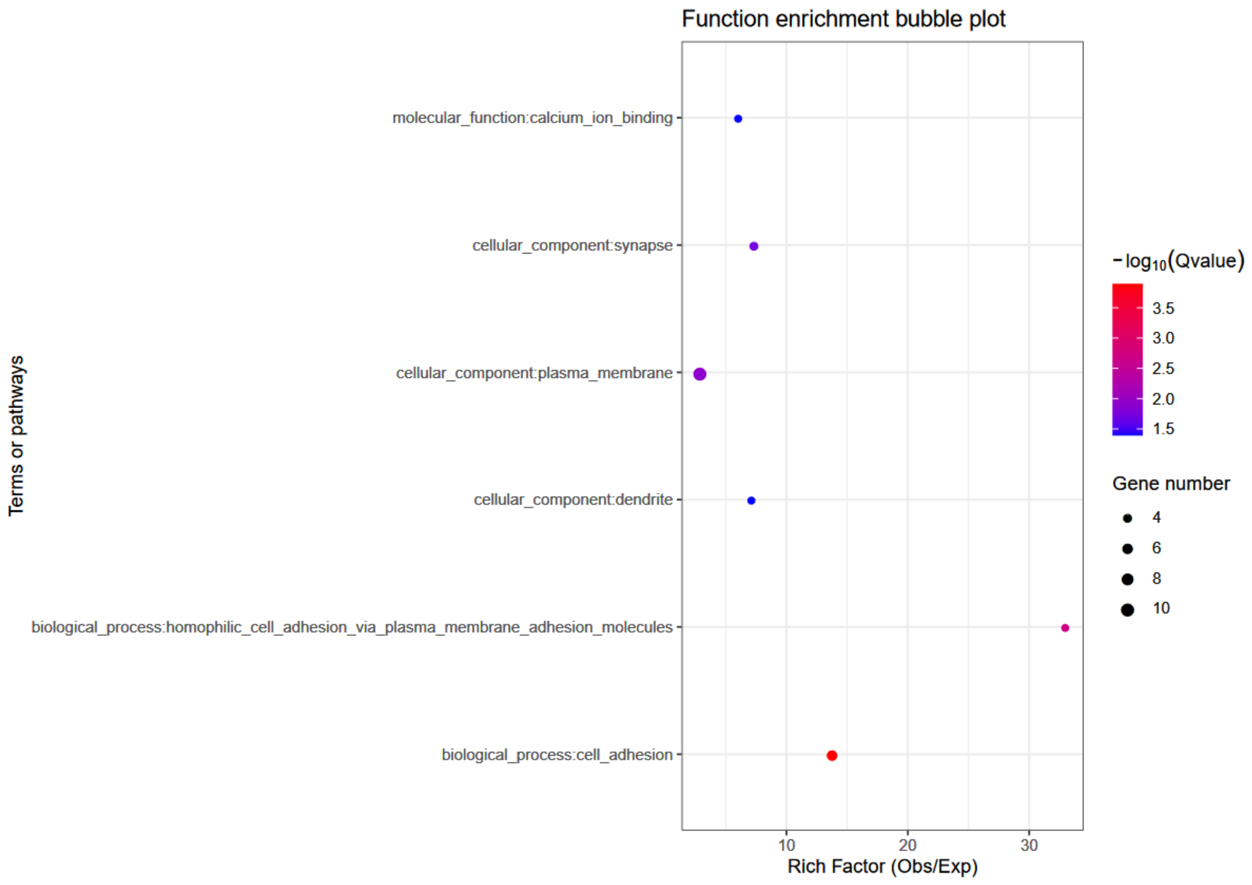

Figure S2a. Bubble plot for GO annotation of 53 Down regulated DEGs is comparison between ADM mice Vs ADM mice+C3Ggroups

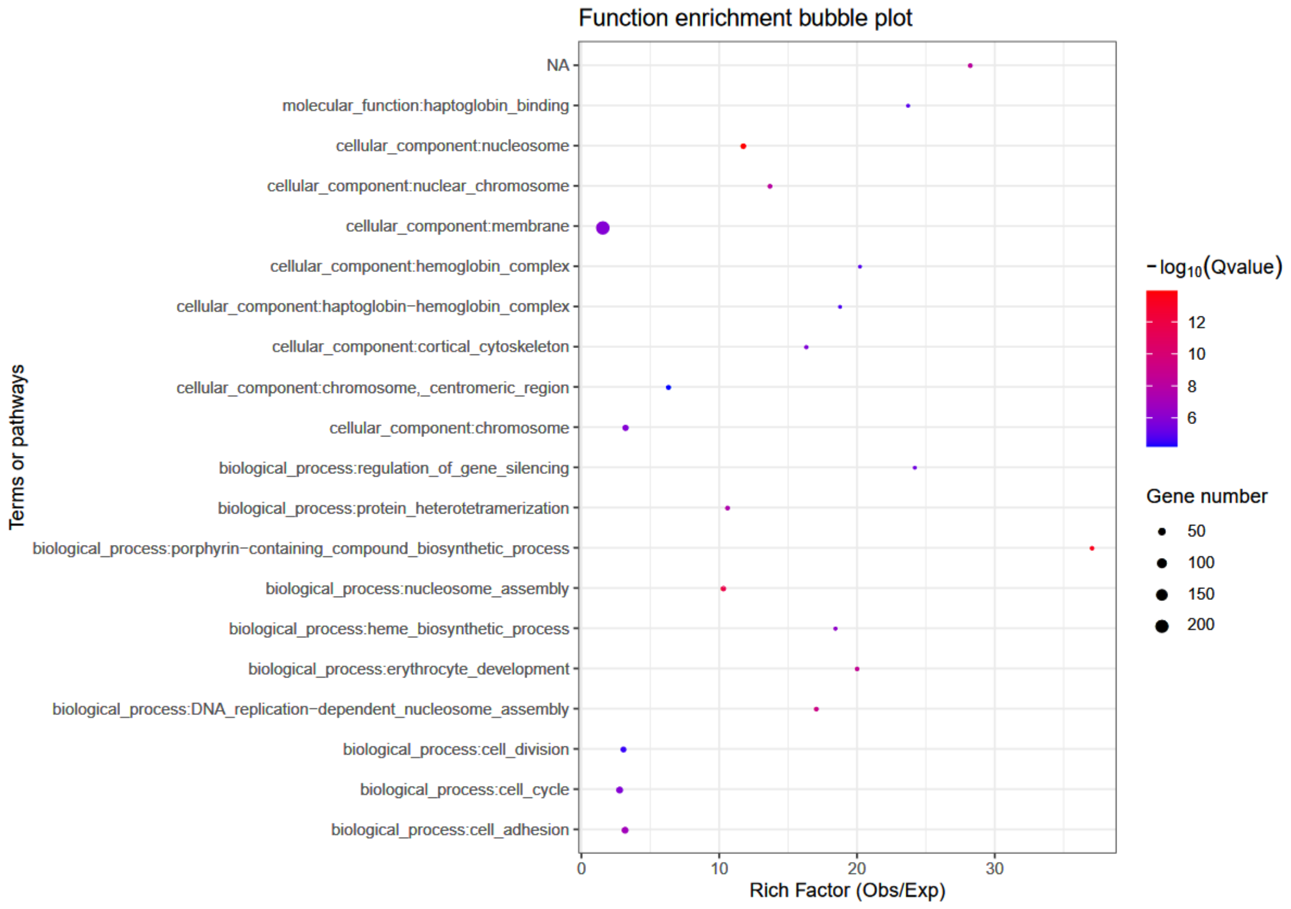

Figure S2b. Bubble plot for GO annotation of 904 Down regulated DEGs is comparison between ADM mice Vs Wt mice groups

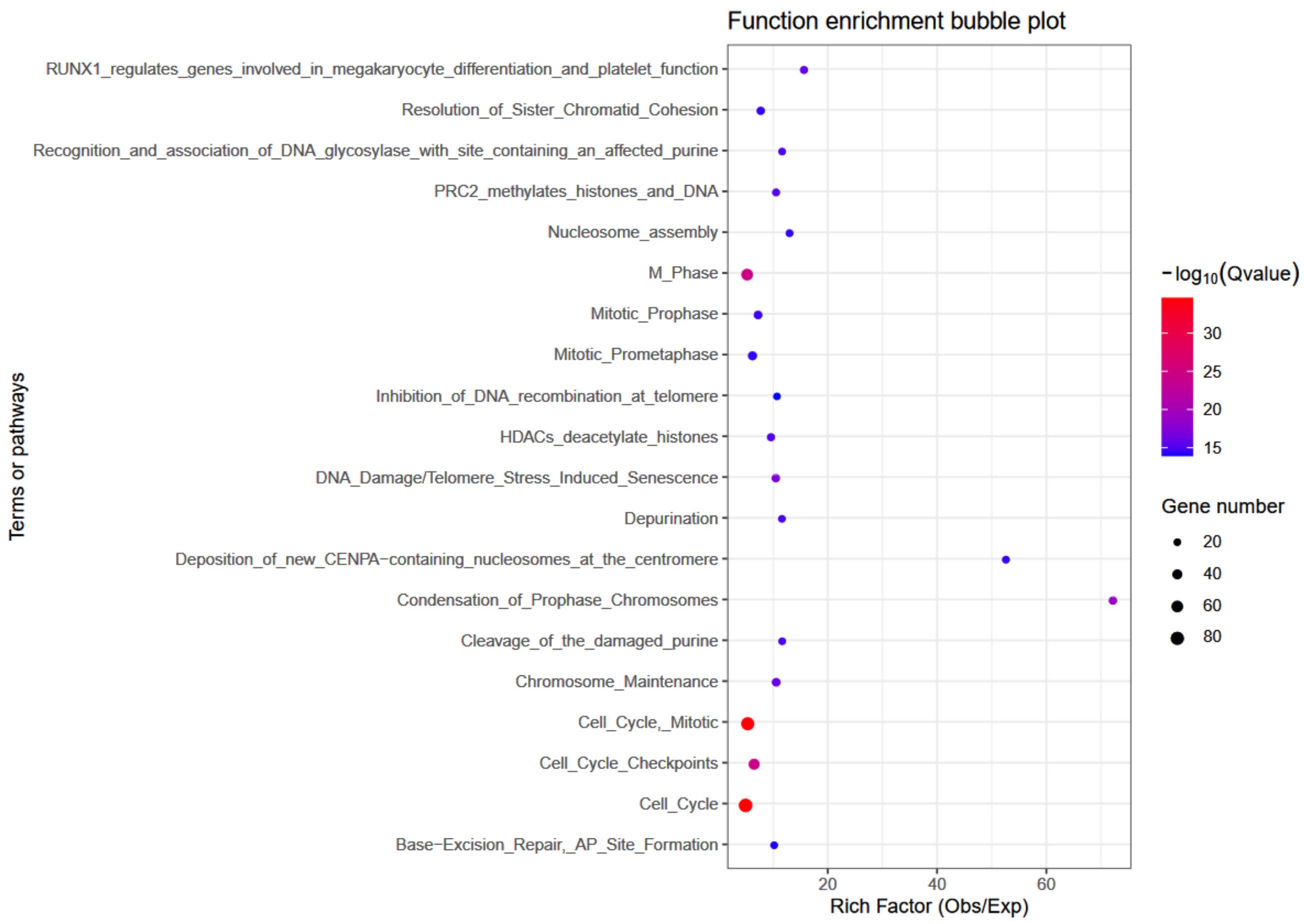

Figure S2c. Bubble plot for reactome annotation of 487 upregulated DEGs is comparison between ADM mice Vs ADM mice+C3G groups

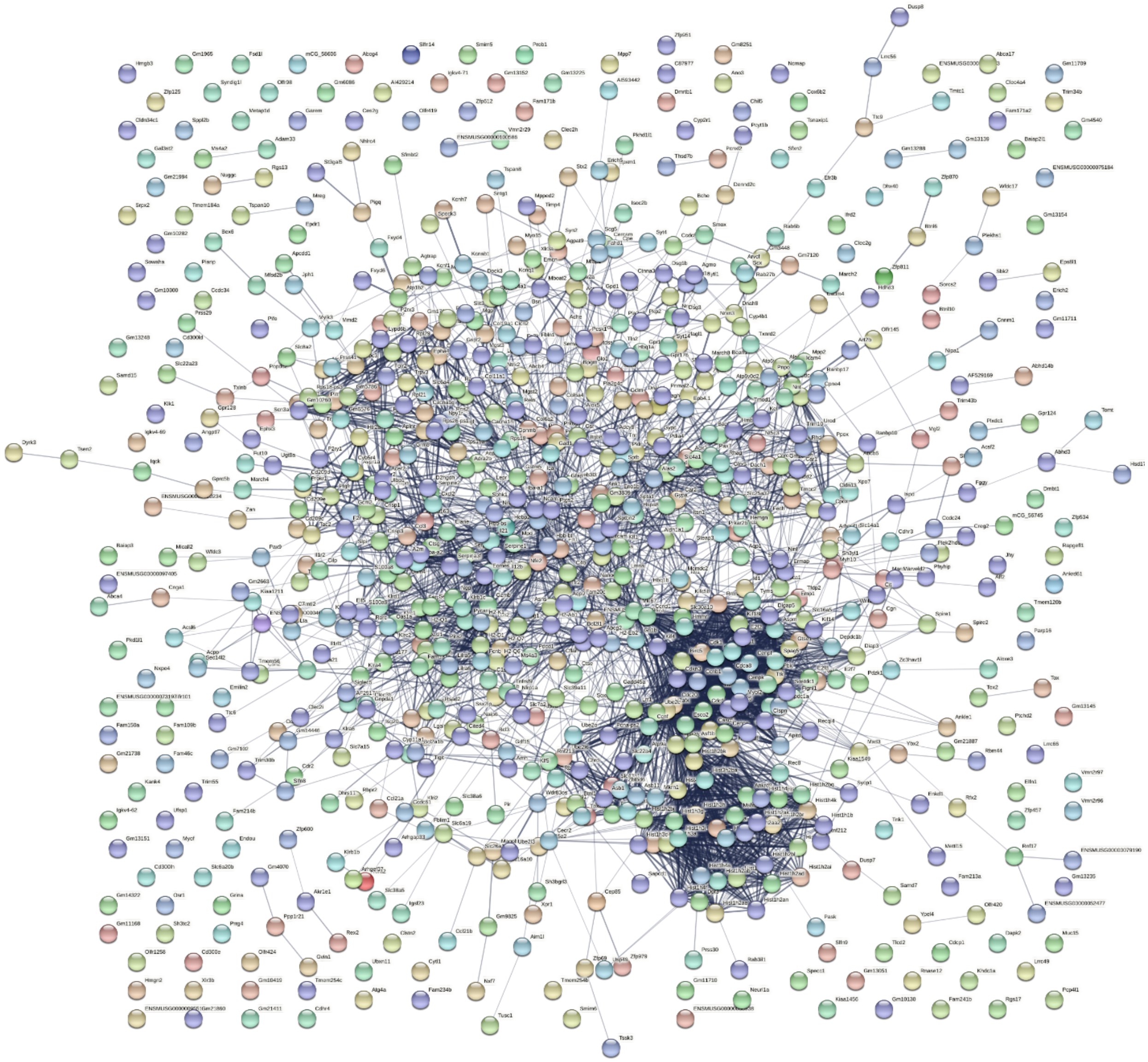

Figure S3a. Protein-protein interaction network between the DEGs identified in ADM mice Vs Wt micecomparison

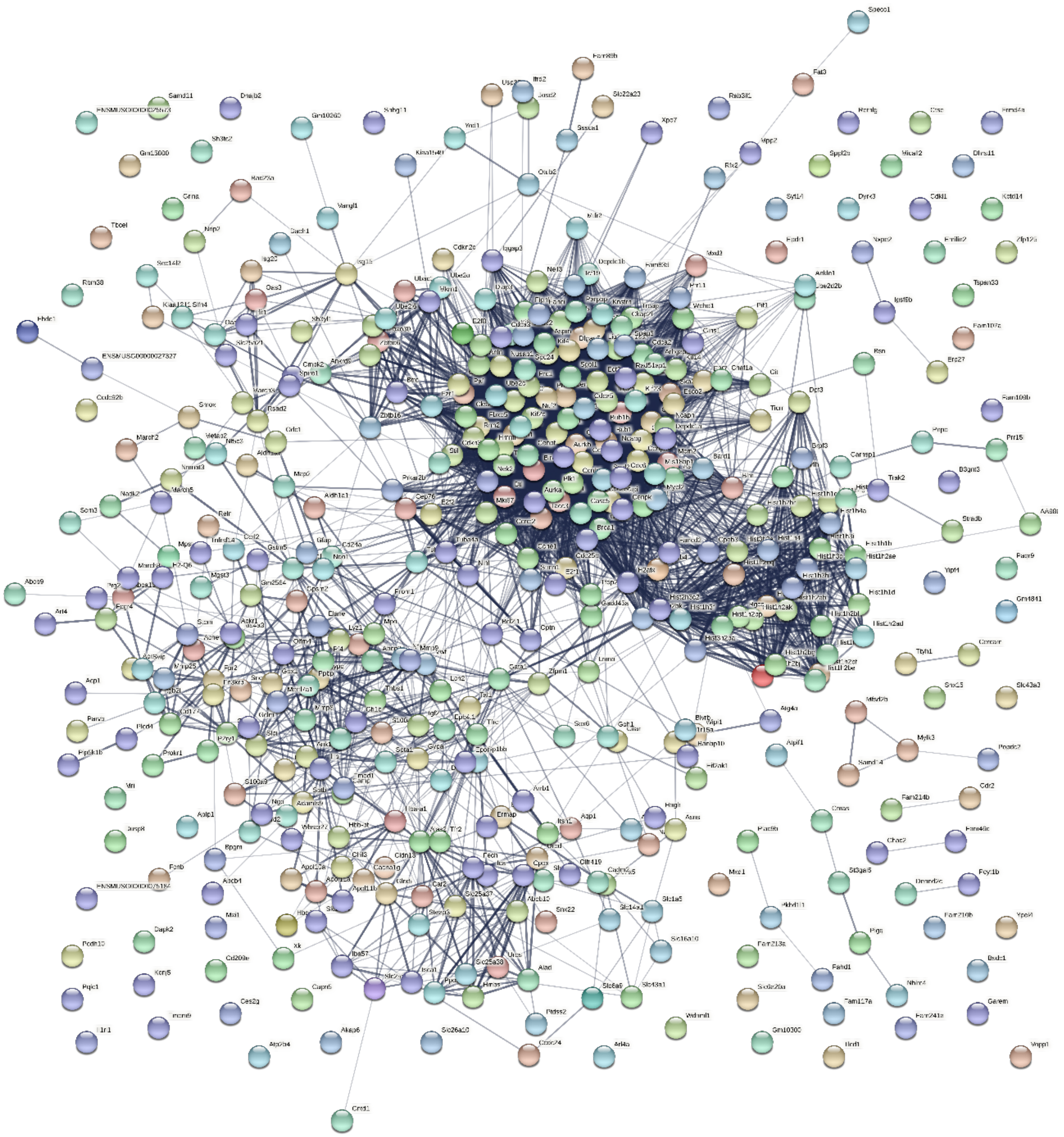

Figure S3b. Protein-protein interaction network between the DEGs identified in ADM mice Vs ADM mice+C3G comparison
